# 3D-Printed Coating of Extended-Release Matrix Tablets: Effective Tool for Prevention of Alcohol-Induced Dose Dumping Effect

**DOI:** 10.3390/pharmaceutics13122123

**Published:** 2021-12-09

**Authors:** Barbora Skalická, Kevin Matzick, Alena Komersová, Roman Svoboda, Martin Bartoš, Luděk Hromádko

**Affiliations:** 1 Department of Physical Chemistry, Faculty of Chemical Technology, University of Pardubice, Studentská 95, 532 10 Pardubice, Czech Republic; barbora.skalicka@student.upce.cz (B.S.); alena.komersova@upce.cz (A.K.); roman.svoboda@upce.cz (R.S.); 2 Department of Analytical Chemistry, Faculty of Chemical Technology, University of Pardubice, Studentská 95, 532 10 Pardubice, Czech Republic; martin.bartos@upce.cz; 3 Center of Materials and Nanotechnologies, Faculty of Chemical Technology, University of Pardubice, Studentská 95, 532 10 Pardubice, Czech Republic; ludek.hromadko@upce.cz

**Keywords:** hot melt extrusion (HME), 3D printing and coating, dissolution testing, alcohol-induced dose dumping effect, Affinisol, polyvinyl alcohol

## Abstract

Tablets used for extended drug release commonly contain large amounts of drugs. The corresponding drug release mechanism thus has to be well-known and invariable under numerous conditions in order to prevent any uncontrolled drug release. Particularly important is the stability and invariability of the release mechanism in the presence of alcohol due to the possible occurrence of the dose dumping effect. The effect of 3D printing (3DP) coating on the drug release mechanism and the drug release rate was studied as a possible tool for the prevention of the alcohol-induced dose dumping effect. Three types of matrix tablets (hydrophilic, lipophilic, and hydrophilic-lipophilic) were prepared by the direct compression method and coated using 3DP. The commercial filament of polyvinyl alcohol (PVA) and the filament prepared from hypromellose by hot melt extrusion (HME) were used as coating materials. Both coating materials were characterized by SEM, DSC, Raman spectroscopy, and PXRD during particular stages of the processing/coating procedure. The dissolution behavior of the uncoated and coated tablets was studied in the strongly acidic (pH 1.2) and alcoholic (40% of ethanol) dissolution media. The dissolution tests in the alcoholic medium showed that the Affinisol coating was effective in preventing the dose dumping incidence. The dissolution tests in the acidic dissolution media showed that the Affinisol coating can also be useful for the delayed release of active substances.

## 1. Introduction

In recent years, the technology of 3D printing (3DP) has significantly affected development in many areas of science and research. The 3DP is a computer-driven process, creating objects in three-dimensional space by deposition of material based on a digital model [1]. For the pharmaceutical research and industry, the method of 3DP represents a challenge not only for an individualized therapy, as is frequently discussed, but also for an improvement of the properties of some types of the solid dosage forms. This study is focused on a coating of matrix tablets with extended drug release using the 3DP in order to increase the resistance of these tablets with respect to alcohol.

It is generally known that alcohol can change or totally destroy the drug release mechanism, which can result in an immediate and uncontrolled release of the active pharmaceutical substance (API) from the dosage form [2,3,4]. This process is called the alcohol-induced dose dumping (ADD) effect. The ADD effect represents a serious problem, especially for controlled release formulations, because these dosage forms contain a high total amount of API in order to reduce dosing frequency. The ADD effect was firstly described in 2005. In July 2005, the Food and Drug Administration (FDA) asked the producer of the drug Palladone™ (capsules with extended release of hydromorphone hydrochloride) to withdraw the product for safety reasons. Taking Palladone™ together with alcohol fatally increases the plasma concentration of hydromorphone, which may depress or stop breathing, cause coma, and even cause death [5,6,7]. In 2009, the FDA gave recommendations for the dissolution studies of controlled release dosage forms containing opioid or non-opioid drugs with a narrow therapeutics range. For the dissolution test, it is recommended to use alcoholic dissolution media with up to 40% of ethanol [6,7,8]. The effect of alcohol on the drug release from oral dosage forms can be reduced using a coating of the dosage form by an alcohol-resistant material. At this time, the literature reporting on the coating of matrix tablets using the 3DP is scarce. Okwuosa and co-workers [9] presented the method for the fabrication of gastric-resistant 3D-printed tablets using dual 3DP. The tablets had a shell-core structure formed by polyvinylpyrrolidone (core) and methacrylic acid co-polymer (shell). Both filaments (for the core and shell) were prepared using a twin-screw hot-melt extruder. The authors confirmed that the shell thickness ≥0.52 mm is necessary to achieve the core protection in the acidic medium, and they applied this technology for the incorporation of different drugs (theophylline, budesonide, and diclofenac sodium). Algahtani and co-workers [10] published a concept for the use of the extrusion-based 3DP technology to print a coating of various designs to modify the drug release from immediate release tablets. They applied the coating system from cellulose acetate in the form of the 3D-printed encapsulating shell for the coating of the tablets containing propranolol hydrochloride. The aim of the work of Tsintavi and co-workers [3] was to partially coat tablets with Precirol ATO 5 using a semi-solid 3D printer and, in this way, to modify the release of two APIs: the hydrophilic methyl-levodopa (Melevodopa) and lipophilic Acyclovir. Their work demonstrated that glycerides can be used for the partial coating of tablets to modify the release rate of the two above-mentioned drugs, without the need to change the tablet composition.

As mentioned above, this study focused on using the 3DP for the coating of tablets with extended drug release to reduce the ADD effect. Two polymers were used as the coating materials: the polyvinyl alcohol (PVA) and hypromellose (HPMC). PVA is a synthetic polymer, which is semicrystalline, non-toxic, water-soluble, insoluble in most organic solvents, biodegradable, and biocompatible in human tissues and fluids [11,12,13,14,15,16]. PVA is able to swell upon contact with aqueous solutions. Depending on the degree of hydrolysis of acetate groups, its melting point may range from 180 (partially hydrolyzed) to 228 °C (fully hydrolyzed) [17]. HPMC is a hydrophilic polymer widely used in pharmaceutical dosage forms, including immediate-release and modified-release formulations [18]. It can contribute to the solubility enhancement of poorly soluble drugs by maintaining the stable solid dispersions and inhibiting API crystallization [11,17]. HPMC can also be used for 3DP. HPMC is an amorphous polymer, which exhibits a broad range of glass transition temperatures (160–210 °C) and a comparatively low decomposition temperature in the range of 200–250 °C. These thermal properties, together with the high viscosity of melt, make it difficult to process the HPMC by hot-melt extrusion (HME) [19,20]. For the preparation of solid dispersions in the pharmaceutical industry by spray-drying or hot melt extrusion (HME), HPMC under the trademark Affinisol^TM^ (AFF) is recommended. Affinisol^TM^ has significantly better properties for processing by HME in comparison with raw HPMC powder [16].

In this work, PVA and HPMC were used in combination with 3DP for the coating of hydrophilic, lipophilic, and hydrophilic-lipophilic formulations of extended release matrix tablets. The commercially available PVA filament and the HME-prepared HPMC filament were used as the coating materials. Differential scanning calorimetry (DSC), scanning electron microscopy (SEM), Raman spectroscopy, and powder X-ray diffraction (PXRD) were used to characterize the coating materials. The resistance and dissolution behavior of the coated tablets (containing tramadol hydrochloride as a model drug) were evaluated in an acidic dissolution medium and in a dissolution medium containing alcohol (40% *v*/*v* according to the FDA Decree [21]).

## 2. Materials and Methods

### 2.1. Materials

Kollidon^®^ SR (BASF SE, Ludwigshafen, Germany) and/or glyceryl behenate (Compritol^®^ 888 ATO, Gattefossé, Saint-Priest, France) were used as the controlled release agents forming the matrix systems. Prosolv^®^ SMCC 90 (JRS PHARMA, GmbH & Co. KG, Herzogenaurach, Germany) was used as a dry binder and Kolliwax (BASF SE, Ludwigshafen, Germany) was used as a lubricant. Tramadol hydrochloride (TH) (Sigma Aldrich Chemie GmbH, Darmstadt, Germany) was chosen as a model highly water-soluble drug. The commercial PVA filament (PrimaCreator, Malmö, Sweden) and the filament prepared from HPMC (Affinisol^TM^, DuPont, Wilmington, DE, USA) by hot melt extrusion were used for the 3DP coating of the matrix tablets. 

Redistilled water and chemicals of analytical grade (HCl, NaCl–Lach-Ner s.r.o., Neratovice, Czech Republic) were used for the preparation of the dissolution media and of the standard solution of tramadol hydrochloride. For the preparation of the alcoholic dissolution medium (40% *v*/*v*), ethanol p. a. (Lach-Ner s.r.o., Neratovice, Czech Republic) was used.

### 2.2. Methods

#### 2.2.1. Preparation of the Matrix Tablets with Tramadol Hydrochloride

All formulations, the compositions of which are described in Table 1, were prepared by the direct compression method at the constant compression force of 8 kN. The model drug and excipients were blended in a mixer (RETSCH MM200, Retsch, Haan, Germany). The prepared mixtures were directly compressed using a manual single-punch tablet press (H-62-TRYSTOM spol. s.r.o Olomouc, Czech Republic). The tablets were of a cylindrical shape without facets; the diameter of the tablets was 13 mm and their weight was 0.5 ± 0.0010 g.

For one dissolution test, six tablets with the model drug and one tablet without the model drug (as a blank sample) were used. In the blank samples, the amount of the model drug was replaced with dry binder Prosolv^®^ SMCC 90. 

#### 2.2.2. Preparation of the Filaments for 3D Coating

##### PVA

PVA was purchased as a commercial filament PrimaSELECT™ PVA+ (PrimaCreator, Malmö, Sweden). The diameter size of the filament was 1.75 mm. The producer recommends a printing temperature of 180–210 °C and bed temperature of 80–90 °C [22]. The filament was used as the coating material without any modifications.

##### HPMC

The HPMC filament was prepared by the hot melt extrusion (HME) process, using the extruder Noztek Pro (Great Britain) with a 1.75 mm nozzle. The HPMC filament was extruded from powdered Affinisol^®^ HPMC HME 15 LV (AFF). For the neat polymer, the producer recommends the lowest processing temperature of 135 °C and the highest processing temperature of 190 °C [23].

Firstly, the extruder was pre-heated to 130 °C for one hour. After that, a batch of 25 g of AFF was gradually added (by about a quarter of the batch) till the hopper was saturated with the material. Next, the temperature of the extruder was gradually increased by 5 °C steps (to a potential maximum of 145 °C), while adding the material to the hopper, until the filament emerged from the nozzle. After some time, a steady flow of filament started to occur at a constant speed. It was possible to take (spool) the filament out of the nozzle manually owing to its sufficient toughness and elasticity. Note that this finding is also essential for the trouble-free implementation of the filament into the 3D printer. Before the 3D printing, the filaments were stored in closed polyethylene (PE) bags at laboratory conditions.

#### 2.2.3. 3D Coating of the Matrix Tablets

**Figure 1:**
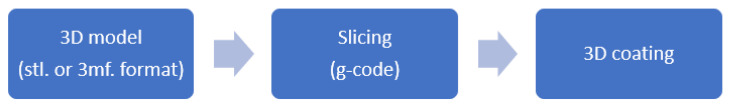
Partial steps of the 3D coating process.

The new strategy of tablet coating with the usage of a 3D printer Prusa i3 MK3 (Prusa, Prague, Czech Republic), the 3D coating, was developed. The whole process is schematically introduced in Figure 1. Once the filaments were prepared, it was necessary to design a 3D model that perfectly fits onto the matrix tablet prepared before. In this case, it was the shape of a cylinder without any infill (only the first two and the last two layers forming the bases were infilled). The basic cylindrical shape was designed in the 3D software Autodesk Fusion 360^®^.

Consequently, the model was imported into the PrusaSlicer^®^ software, where it was also possible to additionally adjust the parameters of the cylindrical object to exactly correspond with the currently coated matrix tablet. Except for the geometric parameters, the printing conditions (temperature of the bed and nozzle, speed, and acceleration of the printing head) were also set in this step.

**Figure 2:**
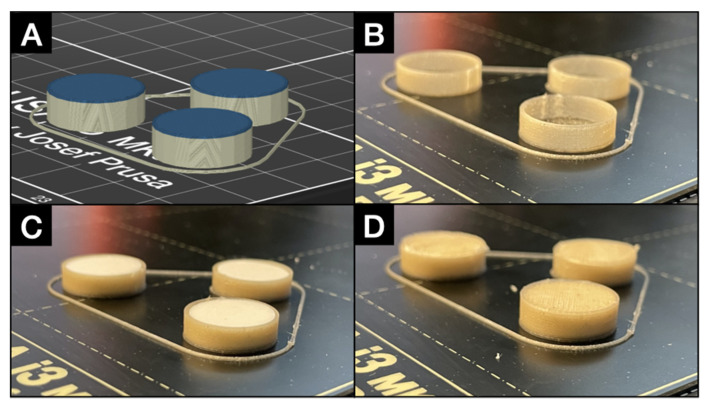
Comparison of the sliced model of the coating (**A**) and the most important steps of the 3D printing process with usage of the PVA filament: (**B**) Moment of the 3D printing interruption. (**C**) Insertion of the matrix tablet. (**D**) Continuation of the 3D printing process and obtaining of the 3D coated matrix tablet.

Each model was sliced into 27–30 layers (4.1–4.5 mm overall height of the cylinder) depending on the matrix tablet height, where the thickness of the first layer was 0.2 mm and the thicknesses of the other layers were 0.15 mm. The height of the cylindrical 3D model was adjusted to the precise height of the matrix tablet. During this step, the operation pause of the process was also added (see Figure 2A; the color change of the layers indicates the moment of stopping the 3D printing process), which enabled insertion of the matrix tablet. 

Finally, the whole sliced 3D model and conditions of the 3D printing process were exported into the g-code, a file that the 3D printer can directly utilize, and that gives the information about the topology of the object and other conditions for the printing process. Once the g-code is exported, it cannot be changed.

The whole 3D coating could thus be understood as the common 3D printing process modified by interrupting the 3D print before the last two layers, so that the tablet could be inserted inside the printed envelope (see Figure 2A,B). After the insertion, the 3D printing process continued with the last two layers (bases) and the 3D coated matrix tablet was obtained. It was important to monitor the dimensions of the coating and the inserted tablet for both to form a continuous upper surface after the tablet insertion (see Figure 2C,D). A tablet protruding out of the coating after the insertion would often cause failure of the reestablished printing meant to 3D print the upper base (“lid”) of the coating.

The present study was focused on two types of filaments: PVA (the commercial one) and AFF (self-prepared by HME). Each material of filament required different conditions for the successful 3D printing, see Table 2.

#### 2.2.4. Scanning Electron Microscopy (SEM) 

The compact scanning electron microscope VEGA3 SBU (Tescan, Brno, Czech Republic) was used to characterize the HME-prepared HPMC filaments, the commercial PVA filament, the 3D-printed coatings made from the two types of filaments, and the Affinisol^®^ powder (HPMC HME 15 LV; AFF). Before the measurement, the samples exhibiting low electrical conductivity were gold-coated by vacuum deposition. These samples were observed under high vacuum at the acceleration voltage of 20 kV, using the detector for secondary electrons. In case of the samples for which the gold coating was not necessary, the backscattered electron detector and the low vacuum mode (10 Pa, N_2_) were used. In general (except for the AFF powder), both the surface and cross-section SEM images were always made for the PVA and AFF filaments as well as for the coatings 3D printed from these filaments. The cross-section cuts were done using a scalpel. The chemical analysis was performed using the SEM-integrated energy-dispersive X-ray microanalysis system Quantax (Bruker Nano XFlash^®^ Detector 410-M and software Quantax Esprit 1.9; Bruker Nano GmbH, Berlin, Germany).

#### 2.2.5. Differential Scanning Calorimetry (DSC)

A differential scanning calorimeter Q2000 (TA Instruments, New Castle, DE, USA) based on heat flow design was used to characterize the coating materials (HPMC and PVA) applied in the present study. The calorimeter was equipped with an autosampler and refrigerating cooling accessory, enabling the measurements from −90 °C. Calibration of the calorimeter was performed via utilization of In, Zn, and H_2_O melting temperatures and enthalpies. All samples were measured hermetically sealed in low-mass aluminum pans, which in fact ensured exposition to static air atmosphere during the measurement (note that the DSC cell was still purged with a continuous 50 cm^3^·min^−1^ N_2_ flow to ensure the steady conditions and to minimize the fluctuation of thermal gradients). The sample masses were approximately 5 mg. The samples were measured in the native form, with no additional treatment, i.e., either in the powdered form or directly cut off from the extruded/printed filament. The samples’ characterization was performed by means of a single heating scan at 20 °C·min^−1^ in the 0 to 275 °C temperature range. Where necessary, the physically meaningful progressive tangential area-proportional baseline was used to approximate the thermokinetic background.

#### 2.2.6. Raman Spectroscopy

The structure of the coating materials (HPMC and PVA) was investigated by means of the Raman spectroscopy. The Raman shift signals were measured using the DXR2 Raman microscope (Nicolet, Thermo Fisher Scientific, Waltham, MA, USA), utilizing the 785 nm excitation diode laser and CCD detector. The applied laser power was 25 mW; each Raman spectrum was obtained as a combination of 100 scans, where the duration of one scan was 5 s. Several Raman spectra were taken for each sample at different spots to confirm the spatial homogeneity (under the 10× magnification and 25 slit collector arrangement the laser was focused at the spherical area with a diameter of 1.6 µm). No baseline corrections were applied.

#### 2.2.7. Powder X-ray Diffraction (PXRD)

An X-ray diffractometer (XRD, PANAlytical Empyrean) was used to record PXRD diffraction patterns for the determination of the presence of crystals in filaments, 3D-printed coatings, and extrudates. It was an X-ray diffractometer (XRD, PANAlytical Empyrean) with Cu-tube and pixcel 3D scintillator detector in the range of 5–65° 2Theta (step size 0.026°) and Bragg–Brentano geometry. For the powder sample, the standard powder sample holder was used. For 3D-printed samples and filaments, the XYZ stage was used. The programmable divergence slit was used to maintain a constant irradiated length of the sample. 

#### 2.2.8. In Vitro Drug Release Studies

Release of the model drug (TH) from the prepared formulations (coated and uncoated matrix tablets) was studied by the dissolution test method. All dissolution tests were performed according to the European Pharmacopoeia 9th (Ph. Eur., European Pharmacopoeia, 2017) using the rotating basket apparatus (Sotax AT 7 Smart, Allschvwill, Switzerland). Two different dissolution media were used: (1) an acidic medium pH 1.2 (HCl with the addition of NaCl prepared according to the Ph. Eur.) and (2) an acidic medium with an addition of ethanol. This alcoholic medium contained 40% *v*/*v* of ethanol and it was prepared from the acidic medium pH 1.2. 

All tests were carried for 18 h at the stirring rate of 100 rpm. The temperature was maintained at 37 ± 0.5 °C. All studied formulations were firstly tested in the non-alcoholic acidic dissolution medium for 18 h. An additional test was performed by starting the dissolution for only 2 h in the alcoholic medium (40% of ethanol in acidic medium pH 1.2; 500 mL) and then transferring the tablets together with the basket into the non-alcoholic acidic medium. Here, the dissolution testing continued for the additional 16 h as described above. 

During the dissolution tests, the samples (3 mL) of the dissolution medium were automatically withdrawn and filtered at predetermined time intervals. The analytical method of UV VIS spectrometry was used for the determination of the TH concentration in the samples. Each experiment was performed with six tablets and one blank sample; the mean values of the released amount of the drug were calculated together with the corresponding standard deviations (SDs). The dissolution profiles obtained in the two types of tests (acidic medium vs. combined alcoholic-acidic medium) were compared. 

##### Mathematical Evaluation of the Dissolution Data

In order to quantitatively evaluate the released amount of the model drug (TH) from the studied coated and uncoated formulations, the time dependences of the released amount of TH were fitted by the first-order kinetic model (Equation (1)) and the Weibull model (Equation (2)). 

For the non-linear regression analysis, the first-order kinetic model was used in the following form:

(1) $$M_{t\left(l\right)}=M_{\infty }\left(1-\exp\left(-k_{1}t\right)\right)$$

where $M_{t\left(l\right)}$ is the amount of the drug released in time *t*, $M_{\infty }$ is the maximum releasable amount of the drug in infinite time (it should be equal to the absolute amount of the drug incorporated into a matrix tablet at the time *t* = 0), and $k_{1}$ is the first order release rate constant expressed with unit *time*^−1^. The Weibull model was applied in the following form:

(2) $$M_{t\left(l\right)}=M_{\infty }\left(1-\exp\left(-k_{w}t^{\beta }\right)\right)$$

where $M_{t\left(l\right)}$ is the amount of the drug released in time *t*, $M_{\infty }$ is the maximum releasable amount of the drug in infinite time, *k*_w_ is a constant of the model with unit *time^-β^*, and a parameter *β* characterizes the shape of the exponential curve. When the shape parameter *β* is equal to one, the Weibull empiric model corresponds to the first-order kinetic model and *k*_w_ corresponds to the first-order release rate constant *k*_1_ [24,25].

#### 2.2.9. Determination of the Released Amount of TH Using UV VIS Spectrometry

A spectrophotometer HP Agilent 8453 was used for the UV VIS determination of the TH concentration in the dissolution samples. The absorbance values of the samples withdrawn at the predetermined times were measured against the corresponding blank samples using the fixed wavelength method (271 nm) with three-point background correction. The used wavelength corresponds to the absorption maximum of our model drug (TH). The validity of the Lambert–Beer law was verified in the expected range of the TH concentrations. To transform the absorbance values into the concentrations and percentage, the calibration curve method was used.

## 3. Results and Discussion

### 3.1. Preparation of the Filaments for 3D Coating

Filaments for the 3D coating are either commercially available (such as PVA filament) or self-made: the hot-melt extrusion (HME) is the most common technology in this regard. This thermal operation was the key point of the whole development of the 3D coatings. The quality of the self-prepared filament influences the process of the 3D printing as well as the physico-chemical properties of the printed coating (mechanical strength, solubility, crystallinity…).

Based on our experience, it is very useful to begin the development with a “pilot extrusion”, the evaluation of the sensoric properties of the obtained filament as a function of the HME temperature. Namely, the lowest temperature at which the filament may occur is of particular importance. At this temperature, the color of the filament is the brightest. On the other hand, the production speed of the filament formation could be quite low. Hence, it is often necessary to find the optimal temperature at which the filament production is acceptable, while the filament is still unburnt. In the case of AFF, the optimal temperature of the HME process was found to be 150–160 °C (as mentioned above, the producer recommends the lowest processing temperature of 135 °C and the highest processing temperature of 190 °C for the neat polymer). Once the “pilot extrusion” is finished, it is possible to start the routine process of extrusion to produce the filament at the conditions of the optimal temperature.

Purging of the extruder is not necessary unless the extruded material is burned and the whole polymer batch needs to be replaced. This process is very time consuming, and the residuals of the cleaning agents may cause further impurities. In case of the purging being necessary, Soluplus^®^ was used as a purging agent. In case of a clogged extruder, it needs to be disassembled at higher temperatures (150–200 °C) and mechanically cleaned. Usage of liquid solvents for the cleaning is not recommended due to the danger of chemical degradation of the extruder components (nozzle, screw, barrel). For each extrusion of new material, it is recommended to use surplus material because the initial section of the produced filament is usually contaminated (often to a visually recognizable extent) with the residuals of the cleaning agents or other impurities.

With regard to the insertion of the filament into the 3D printer, the mechanical properties of the prepared filaments were also optimized. The filament had to be durable enough, but not too smooth, because then the gears of the filament feeder were slipping, and the filament could not be utilized despite its best qualities. 

### 3.2. 3D Coating of the Matrix Tablets

The 3D coating model was firstly designed using the software described in Section 2.2.3. As the commercial PVA filament has the recommend temperatures ranges of 180–210 °C for the nozzle and 80–90 °C for the bed [22,26], the best results were achieved for 190 (nozzle) and 80 °C (bed). Higher temperatures caused the print deformation due to material softening. It was also beneficial to prepare the 3D model consisting of several coatings at once, see Figure 2, where 3 coatings were printed at once. In this way, the layers had enough time to solidify before the next layer was added. On the other hand, if this delay was too long, the cohesion of each single layer was affected, and the 3D-printed model was unstable and brittle (very often damaged during removal from the bed).

In the case of the self-prepared AFF filament, the best results were achieved for the temperatures of 205 °C at the nozzle and 90 °C at the bed. AFF coatings were quite brittle; therefore, careful manipulation during the insertion of the matrix tablets was important. The best method for the removal of the 3D coated tablet from the bed was found to be the following: cooling the bed, lifting the magnetic layer of the bed (with the 3D-printed coatings on its top) and its bending, and careful picking up of the coatings using a spatula or a 3D-printed rectangular-shaped object (credit card shape, 0.2 mm thin, made of poly-lactic acid).

**Figure 3:**
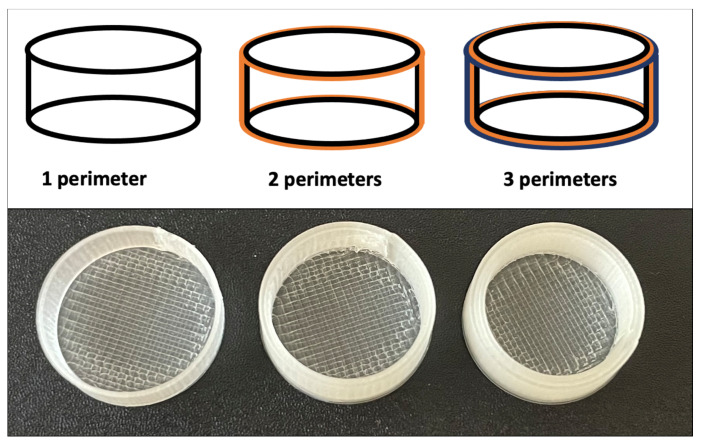
Schematic representation of 3D-printed coatings (PVA) with various perimeters (P1, P2, P3).

The next parameter that was optimized was the number of perimeters (P), which determines the thickness of the coating (see Figure 3). The 3D coatings were prepared in three versions according to the number of printed perimeters: P1, P2, and P3. For the fixed diameter of the coating, the free volume will decrease with an increasing number of perimeters, which would make the insertion of the tablet impossible (see Figure 3). Therefore, the outer diameter of the coating had to be adjusted for each perimeter number, so that the inner diameter of the coating remained constant, optimized for the exact matrix tablet size. With a nozzle diameter of the 3D printer of 0.4 mm, the following outer diameters of the P1, P2, and P3 coatings were used: 14.4, 15.2, and 16 mm, respectively. 

The 3D coating times depending on the number of coatings per 3D printing and their perimeters are summarized in Table 3. The main benefit is that it can be prepared in advance, whereas the steps of insertion of the matrix tablets and completion of the 3D coating process follow. This can save much more time, because the final 3D coating step represents only ca. 25% of the overall times.

According to our experience, it is recommended to start with a lower number of coatings for each process in order to test the stability of the process. In case of the PVA filament, it is not recommended to 3D print single coatings, because the layers do not have enough time to solidify and therefore the whole coating could be deformed. These issues did not occur in the case of the AFF filaments.

It can also be seen that increasing the 3D coating thickness (number of perimeters) is undemanding, reproducible, and does not require much extra time. However, it is important to bear in mind that the coating thickness is directly related to the nozzle diameter of the 3D printer, and it is not possible to prepare thinner coatings than this value. 

In conclusion, the most critical parts of the 3D coating development were as follows:

- Preparation of the filament: choice of materials (or their combinations) that can melt and form a stable filament with required mechanical properties (elasticity and toughness).
- Ability to insert the filament into the 3D printer: successful preparation of the filament by HME does not guarantee the insertability into the 3D printer. Too fragile and smooth filaments are not utilizable in this regard.
- Mutual cohesion of the printed layers during (and also after) the 3D printing process. If the cohesion is insufficient, the model is unacceptably fragile.

### 3.3. SEM

The preliminary macroscopic examination of the materials showed that the commercial PVA filaments (both before and after 3D printing) are visually glassy and translucent-to-opaque white. The AFF filaments showed a rather brownish opacity. The PVA filaments were more flexible, whereas the AFF filaments had a stronger shape memory and higher hardness. 

**Figure 4:**
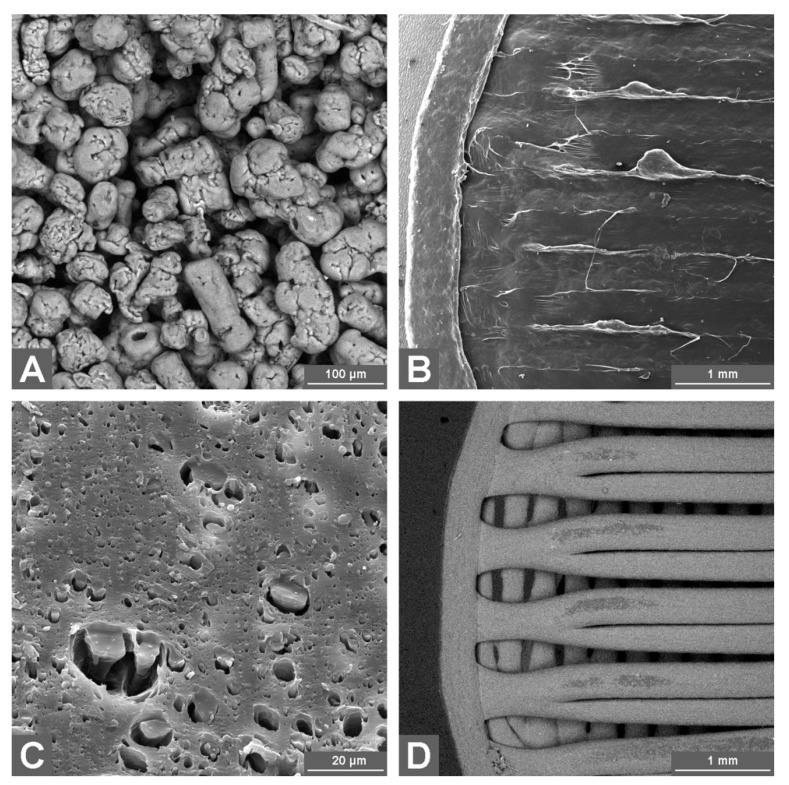
Representative SEM images of the AFF powder (**A**), detail of the AFF coating (**B**), detail of the cross-section area of the PVA filament (**C**), and detail of the PVA coating (**D**).

From the microscopic (SEM) point of view, the majority of the **AFF powder** was composed of particles with ovoid-to-spherical characters, usually less than 100 μm in diameter, with a shape reminiscent of extruded corn. A minor part of the AFF powder was formed by compact cylindrical structures with an approximate diameter of 20 μm and length of 100 μm (Figure 4A). Rare but clearly visible were the cuboid crystals of NaCl-type less than 100 μm in size. Note that during the extrusion and 3D printing, similar crystals were observed, forming rare clusters on the filament surface. No cuboid crystals were detected inside the filaments.

The **AFF filament** was found to have a smooth surface, with traces of the extrusion process detectable at higher magnifications. The observed surface erosion was in the form of burrs and discontinuous fine lines parallel to the filament axis. The origin of the burrs might possibly be associated with the tearing of the cooling material from the nozzle during the filament’s travel through the narrowed opening. Few small cavities were present on the surface. The filament has a circular cross-section, where the absolute majority of the material is compact with only few isolated cavities (diameter up to 1 μm). 

The 3D-printed **AFF coating** consists of two parts (circular bases and cylindrical wall), differing in their shape and properties. The wall is formed from flat AFF rings mounted on each other (Figure 4B). The links between the rings are quite weak, breaking under even slight mechanical stress. The bases are formed from two layers of AFF fibers. The fibers in each layer are mutually parallel; the fibers in the upper layer were laid perpendicular to the fibers of the bottom layer. These walls were compact and mechanically resilient, without holes and gaps. The interconnections between the bases and the cylindrical wall were similar to those between the wall rings, easily separable by mechanical stress. The AFF coating is thus fully enclosed with no openings. The dry coating is, however, mechanically only a little resilient.

The **PVA filament** has a smooth but uneven surface, as if shallow dentation happened. Alternatively, the uneven surface could be caused by volume contraction that occurred during the cooling, the cause of which could be the temperature-related drop in the pressure of the gas bubbles trapped in the filament volume. The filament has a circular cross-section and is highly porous, containing a large number of isolated cavities with diameters varying from the detection limit up to 20 μm. The total area of the cavities can be estimated at 10–20% of the cross-section area (Figure 4C).

In case of the **PVA coating**, the individual layers of the cylindrical wall hold together well even under mechanical stress. The circular bases have a similar structure as those made from AFF: two layers, parallel fibers within each layer, and fibers in the upper and bottom layers rotated by 90° with respect to each other. Owing to the larger gaps in-between the individual fibers, the structure of the bases is similar to a sieve. The largest gaps were found at the edges of the bases. Hence, the walls are permeable for the dissolution medium before the swelling of the PVA fibers. After the 3D printing, the material is similarly porous to the PVA filament, only the cavities are more spherical, with a diameter ~2 μm (Figure 4D).

### 3.4. DSC and Raman Characterization

**Figure 5:**
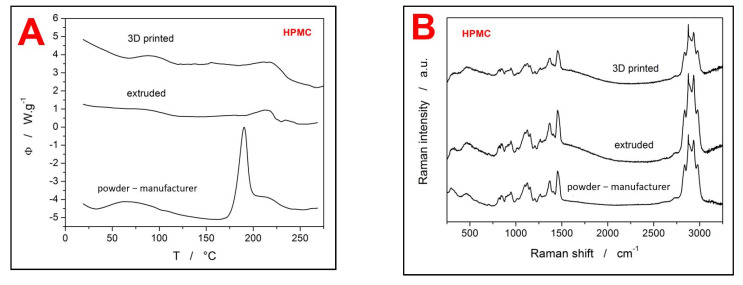
(**A**) DSC traces of the as-purchased (powdered) HPMC, extruded HPMC filament, and 3D-printed HPMC structure. The exothermic effects evolve in the upwards direction. (**B**) Raman spectra of the three studied HPMC forms.

Differential scanning calorimetry and Raman spectroscopy were used to characterize the coating materials (HPMC and PVA) in order to reveal their potential changes throughout the processing setup. In case of HPMC, three stages of the material treatment were compared: the as-purchased powdered HPMC (particle size distributions are D(0.1) 54.35 µm, D(0.5) 104.49 µm, and D(0.9) 207.07 µm) [14], HPMC filament from extrusion, and 3D-printed HPMC material. The DSC curves obtained for the three HPMC forms at 20 °C·min^−1^ are shown in Figure 5A. There are several features identifiable on the DSC curves. As the sample is heated, the first observed process, associated with a broad endotherm at approximately 30–80 °C, is the release of weakly bound or absorbed water. The water evaporation is particularly pronounced in the case of the powdered as-purchased material, which is, however, expected due to the large surface area. In the case of the extruded filament, the endotherm is practically non-existent, which corresponds to the highly compact nature of this HPMC form. Interestingly, the 3D-printed HPMC sample also shows a significant amount of water being released. The higher water vapor uptake may be the consequence of either the much smaller diameter of the 3D-printed filament (compared to the extruded one) or the morphologically more complex structure of the 3D-printed objects. The second prominent feature on the DSC curves in Figure 5A is the glass transition (endothermic step-like change of the heat capacity) in the ~85–125 °C temperature range, which is in good agreement with the literature [17,23]. As is apparent, both filaments exhibit a much more uniform and more recognizable transition between the glassy and undercooled liquid regions. This is a common sign of the material being fully amorphous. On the other hand, the as-purchased powdered HPMC shows relatively weak and segmented glass transition, which may indicate higher diversity of the structural ordering on the molecular level achieved during the HPMC manufacturing. Nevertheless, all three HPMC forms have a similar value of the glass transition temperature T_g_, which confirms that no significant physico-chemical changes were introduced into the materials’ structure during their processing (extruding, 3D printing). The third feature recognized on the DSC curves is the exothermic peak at circa 180–230 °C, which corresponds to the crystallization of the glassy matrix. This peak is markedly pronounced in the case of the as-purchased powdered HPMC, which indicates its higher tendency toward crystallization. This is understandable due to the much higher specific surface of HPMC in this form, as well as due to the larger amount of mechanically induced defects (formed during the manufacturing) that can act as crystal growth centers. The DSC curves for the two filaments (extruded and 3D printed) show only small crystallization signals, as a consequence of the initially high amorphous with low amounts of nuclei and surface crystallization centers. The lower tendency of the HPMC filaments towards the crystallization is also evidenced by the shift of the crystallization peak onset to the higher temperatures. The enthalpy changes associated with the crystallization of the three HPMC forms were: 157.2, 9.2, and 12.8 J·g^−1^ for the as-purchased, extruded, and 3D-printed HPMC, respectively. Similarly, as in the case of the water uptake, the slightly higher crystallization enthalpy of the 3D-printed HPMC (compared to the extruded filament) also implies its looser structure and defect-containing morphology that is more prone to destabilization. The last prominent signal observed on the DSC curves was the large exothermic peaks corresponding to the thermal decomposition of HPMC [20], although only the onset of this process (at ~275 °C) is shown in Figure 5A.

In addition to the DSC characterization, the Raman spectroscopy was also used to confirm the stability of HPMC during the extrusion and 3D printing procedures. The Raman spectra of all three HPMC forms are shown in Figure 5B. Overall, all three Raman spectra displayed in Figure 5B correspond very well with the literature data on HPMC [27,28]. Additionally, there are no differences between the particular spectra, neither in the positions of the Raman bands nor in their relative intensities.

**Figure 6:**
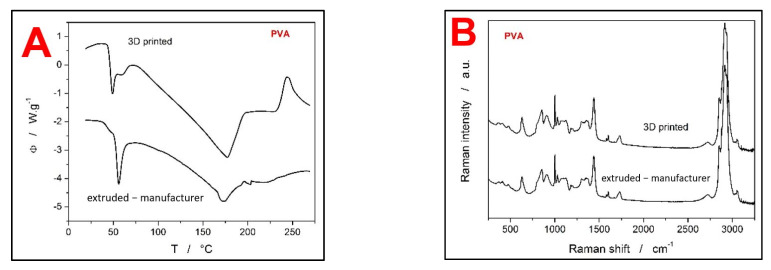
(**A**) DSC traces of the as-purchased extruded PVA filament, and 3D-printed PVA structure. The exothermic effects evolve in the upwards direction. (**B**) Raman spectra of the two studied PVA forms.

The second investigated coating material was the PVA. As this material was commercially available directly in the form of an extruded filament that is ready and optimized for the 3D printing, only two forms of PVA were characterized within the framework of the present study: the as-purchased extruded filament and 3D-printed structure. The DSC curves of the two PVA forms are shown in Figure 6A. No broad low-temperature endotherms are present in the DSC records, which indicates that both filaments exhibit practically no water uptake. However, both PVA forms show a complex glass transition phenomenon, which in addition occurs at significantly lower temperatures (40–68 °C) compared to pure PVA (~81 °C, [29]). This may be caused by the PVA filament containing some impurities (e.g., polyvinyl acetate from the potential hydrolytic PVA production, or ethyl acetate and vinyl esters from the potential base-catalyzed transesterification production of PVA). The second less probable explanation of the complex glass transition could be associated with a significant amount of crystalline being present. The semi-crystalline PVA could then exhibit two separate glass transitions based on the degree of crystallinity attributed to the particular material cluster. Anyway, the 3D printing process has to an extent alternated the ratio between the two overlapping endotherms in the glass transition range, but their position (corresponding to the chemical entity) remained unchanged. Similarly to the glass transition, the melting endotherm of the present PVA samples also occurs at lower temperature (~160–190 °C) compared to the literature data (onset at 200 °C [30]). The PVA melting peak also seems to not be affected by the 3D printing procedure.

Concerning the Raman spectra (see Figure 6B), the records for both measured PVA forms (the as-purchased extruded filament and the 3D-printed structure) were perfectly identical, showing no changes in the positions or intensities of the Raman shift bands. Similarly to HPMC, the obtained Raman spectra of PVA also also correspond with the literature data also [31], indicating that no degradation or alternation of the materials’ molecular structure took place during the processing routines (extrusion, 3D printing).

To conclude, the extrusion and 3D printing of HPMC and PVA polymers were under the present conditions found to produce stable coatings, with the physico-chemical properties similar to those of the source/input materials. No material degradation was detected.

### 3.5. Powder X-ray Diffraction (PXRD)

**Figure 7:**
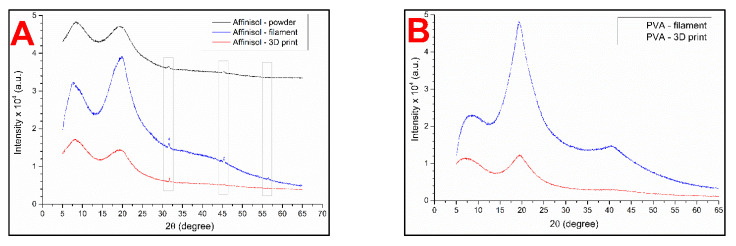
(**A**) Powder X-ray diffraction characterization of Affinisol in different forms (powder, filament, 3D-printed sample) and (**B**) PVA as a commercial filament and 3D printed form.

The powder X-ray diffraction technique was used to determine the potential presence of the crystalline phase in the studied samples: Affinisol powder, Affinisol filament, Affinisol 3D print, PVA commercial filament, and PVA 3D print. The PXRD patterns are shown in Figure 7.

In case of the PVA samples, no characteristic major peaks indicating the presence of a crystalline phase were identified; hence the materials can be considered fully amorphous. In case of Affinisol, small PXRD peaks indicating a minor presence of crystalline material can be found in all samples. However, when compared to literature data, only the halo at 20° 2θ can be considered a signal from the polymeric material (amorphous form of cellulose) [20,29]. It is noteworthy that in many polymers, a small portion of the crystalline phase naturally exists due to the inter- and intra-molecular hydrogen bonds causing ordering of the polymer chains [20]. The PXRD peak at 31.65° 2θ can probably be attributed to the presence of NaCl, which is the main known contaminant of Affinisol. In [28], the presence of NaCl in amorphous Affinisol was confirmed based on the PXRD peak found at 31.09°. A similar explanation can also be adopted for the second minor peak at 45.38° 2θ, which may indicate the traces of sodium carbonate as a second probable contaminant. These findings are also consistent with the conclusions resulting from the SEM characterization. Attribution of the observed PXRD peaks to the presence of high-melting contaminants (inorganic salts) can also be supported by the fact that the relative intensity of the PXRD peaks practically does not change with thermal treatment including multiple heating-cooling cycles, during which the polymer repeatedly melts and solidifies. Under such circumstances, the potential degree of polymer crystallinity should be certainly altered due to the different conditions for each cooling cycle (including the initial production of purchased Affinisol). 

### 3.6. In Vitro Drug Release Studies

The evaluation of changes of the dissolution profiles due to the presence of alcohol was used as a suitable method for testing the alcohol resistance of the uncoated and coated matrix tablets. The dissolution behavior of all studied formulations was studied in the acidic dissolution medium, and these dissolution profiles were compared to the profiles obtained in the presence of alcohol (2 h in 40% alcohol, then 16 h in acidic medium).

This study is primarily focused on the release of the model drug tramadol hydrochloride in the acidic medium containing 40% alcohol. Generally, the change of pH of the dissolution medium can significantly influence the drug and excipients’ solubility resulting in the change of the dissolution behavior. Therefore, the dissolution behavior of all uncoated formulations and formulations with 3D-printed Affinisol coating (one value of perimeter thickness Affinisol I) in a phosphate buffer (pH 6.8) was also studied. The results of these dissolution tests are shown in the Supplementary Materials. A significant change of k_w_ was found for lipophilic formulation F2. As the aqueous solubility of tramadol hydrochloride is >20 mg/mL in the pH range 1.2–7.5 [32], a negligible effect of pH on the TH solubility can be assumed. The decrease of the release rate of TH from F2 may be caused by the change of the solubility of excipients.

#### 3.6.1. Acidic Medium pH 1.2: Dissolution Tests of the Uncoated Tablets and Tablets with PVA Coating

**Figure 8:**
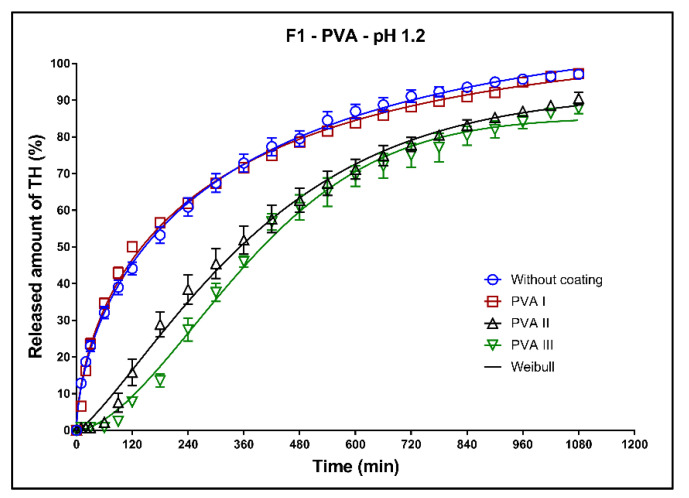
Dissolution profiles of F1 formulation in acidic medium fitted to the Weibull model: uncoated tablets and tablets with PVA coating (three values of perimeter thicknesses).

**Figure 9:**
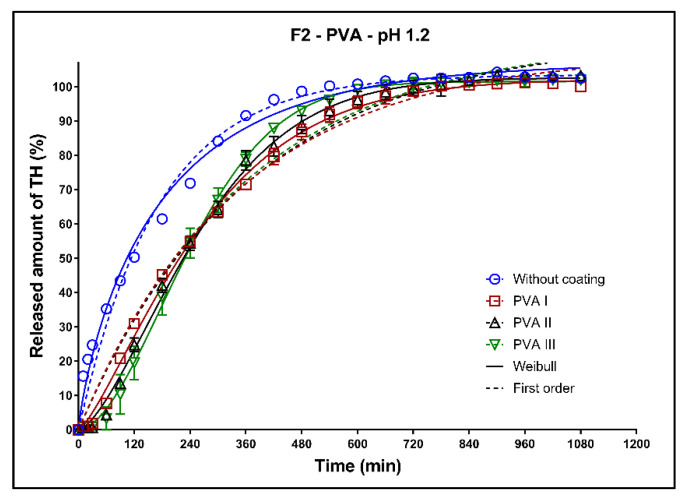
Dissolution profiles of F2 formulation in acidic medium fitted to the first-order kinetic model and Weibull model: uncoated tablets and tablets with PVA coating (three values of the perimeter thickness).

**Figure 10:**
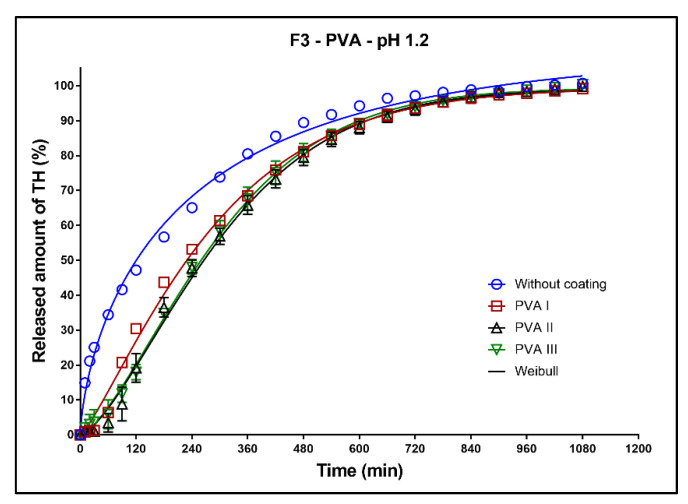
Dissolution profiles of the F3 formulation in acidic medium fitted to the Weibull model: uncoated tablets and tablets with PVA coating (three values of the perimeter thickness).

In Figure 8, Figure 9 and Figure 10, the dissolution profiles of three studied formulations (F1–F3; see Table 1) without coating and with 3D-printed PVA coating (three values of perimeter thicknesses: PVA I, PVA II, PVA III) can be seen. The dissolution profiles were fitted to the first-order kinetic model and the Weibull model. The results of the non-linear regression analysis are summarized in Table 4, Table 5, Table 6 and Table 7. A detailed description of the dissolution behavior follows.

**Formulation F1**: All dissolution profiles were fitted to the Weibull model (Figure 8). The dissolution profile of the uncoated tablets is very similar to the dissolution profile of the tablets with the PVA I coating. The one perimeter thick coating had practically no effect on the drug release, which was also confirmed by the values of *k*_w_ (Table 4). In the initial phase of the dissolution test (3 h), the release of TH was faster than that of the first-order kinetic model. At the end of the dissolution test, 98% of the model drug was released. As can be seen in Figure 8, the greater thickness of the coating (perimeters II and III) caused the delay at the beginning of the dissolution test (lag time). The maximum released amount of the model drug from these coated tablets after 18 h of the dissolution test was slightly lower (difference ~10%) compared to the uncoated tablets.

**Formulation F2**: All dissolution profiles were fitted to the first-order kinetic model and Weibull model. As can be seen in Figure 9, the fitting of the dissolution profile of the uncoated tablets provided very similar results for both mentioned models. With an increasing thickness of the coating, the differences between the first-order kinetic model and Weibull model increase. This fact is a result of the coating resistance and the lag time at the beginning of the dissolution process. The coefficient of determination (*R^2^*) for the first-order kinetic model decreases with the increasing thickness of the coating, but *R^2^* for the Weibull model is almost the same for all thicknesses of the coating (Table 6). At the end of the dissolution tests, 100% of the model drug was released. The profiles of all coated tablets are very similar, and the dissolution behavior of these tablets is minimally affected by the thickness of the coating. 

**Formulation F3**: All dissolution profiles were quantitatively evaluated using the Weibull model (Table 7). As can be seen in Figure 10, during the first 10 h of the dissolution test, the coating strongly slows down the release of the model drug compared to the uncoated tablets, but 100% of the model drug was released during the dissolution test. The dissolution profiles of all coated tablets are very similar. The release of TH from the tablets with coating PVA I was slightly faster in the first 8 h compared to the tablets with coatings PVA II and PVA III. As can be seen in Figure 10, the thickness of the coating does not significantly affect the drug release. These facts were also confirmed by the similar values of *k*_w_ (Table 7).

#### 3.6.2. Acidic Medium pH 1.2:Dissolution Tests of the Uncoated Tablets and Tablets with Affinisol Coating

**Figure 11:**
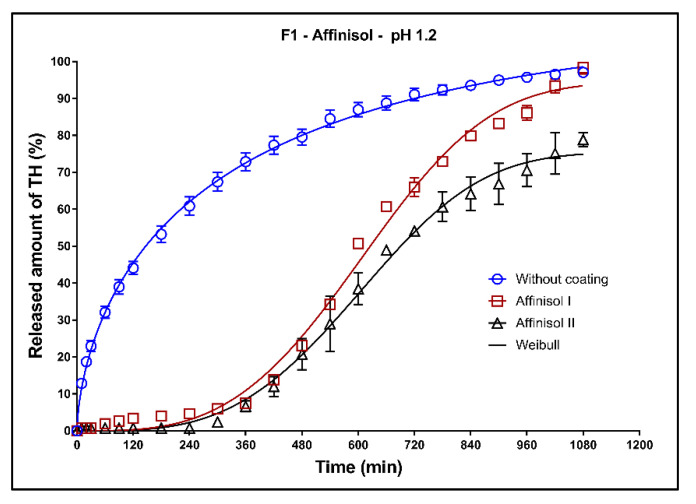
Dissolution profiles of F1 formulation in acidic medium fitted to the Weibull model: uncoated tablets and tablets with Affinisol coating (two values of the perimeter thickness).

**Figure 12:**
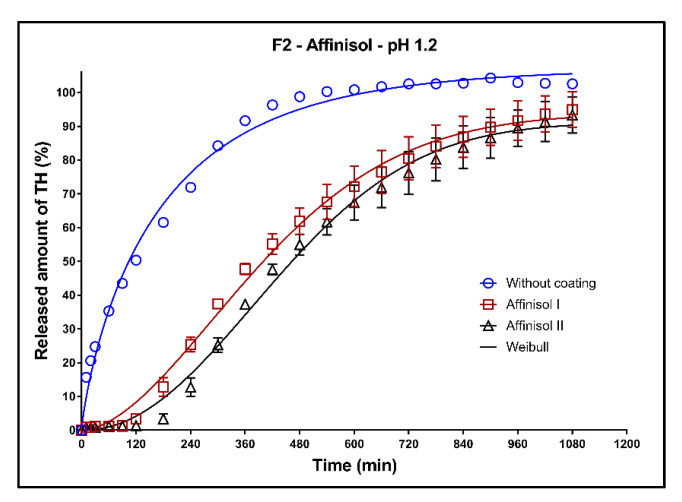
Dissolution profiles of F2 formulation in acidic medium fitted to the Weibull model: uncoated tablets and tablets with Affinisol coating (two values of the perimeter thickness).

**Figure 13:**
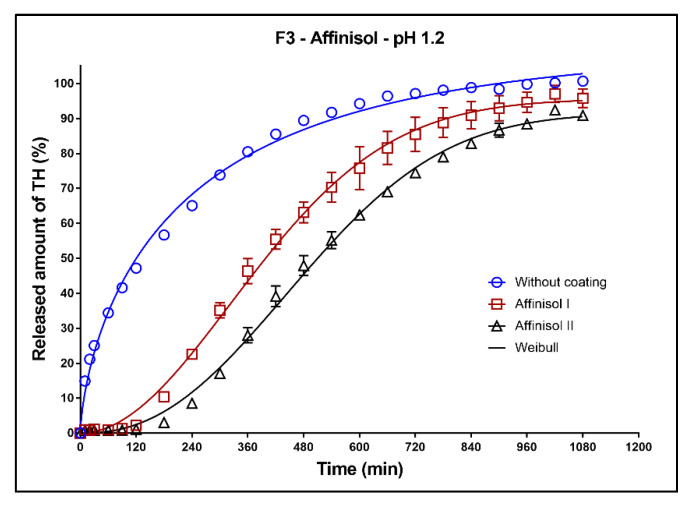
Dissolution profiles of F3 formulation in acidic medium fitted to the Weibull model: uncoated tablets and tablets with Affinisol coating (two values of the perimeter thickness).

In Figure 11, Figure 12 and Figure 13, the dissolution profiles of three studied formulations (F1–F3; see Table 1) without coating and with 3D-printed Affinisol coating (two values of perimeter thickness: Affinisol I, Affinisol II) can be seen. The dissolution profiles were fitted to the Weibull model. The results of the non-linear regression analysis are summarized in Table 8, Table 9 and Table 10. A detailed description of the dissolution behavior follows.

**Formulation F1**: Release of TH from the uncoated tablets is very fast, as can be seen in Figure 11. During the first 3 h of the test, about 50% of the model drug was released. In the case of the coated tablets, a sigmoidal character of the dissolution profiles was found. The maximum released amount of the model drug after 18 h of the dissolution (approximately 98%) was very similar for the tablets without coating and coated tablets with the thickness of coating 1 (Affinisol I). For the coated tablets with 2 perimeters thick coating (Affinisol II), the maximum released amount of TH was lower (approximately 80%). The coating was resistant for 3 h, during which the model drug was not released. This type of formulation and coating would be suitable for delayed drug release. 

**Formulation F2**: Release of TH from the uncoated tablets is very fast (faster than for F1), as can be seen in Figure 12. The coefficient ß of the Weibull model is 0.89 ± 0.03 (Table 9), which means that the drug release follows the first-order kinetics. During the first 2 h of the test, about 50% of the model drug was released. The coating was resistant only for approximately 2 h. 

After 2 h of the test, the barrier for the transport of the dissolution medium through the tablet coating was broken and subsequently the medium began to penetrate into the matrix. The resistance against the dissolution media penetration is better in case of the matrix tablets with Kollidon SR (F1) compared to tablets with Compritol (F2). 

**Formulation F3**: The fit of the dissolution profile of the tablet without coating by the first-order kinetic model and Weibull model were rather dissimilar. The tablets with coating exhibited the S-shaped dissolution profile (Figure 13). The coating of the matrix tablets was resistant against the dissolution media for 1 h. The maximum released amount of the model drug after 18 h was 100% for the tablets without coating. The maximum released amounts for the coated tablets AFF I and AFF II were ~90 and 80%, respectively. 

#### 3.6.3. Alcoholic Medium (40% of Alcohol): Dissolution Tests of the Uncoated Tablets and Tablets with PVA Coating 

**Figure 14:**
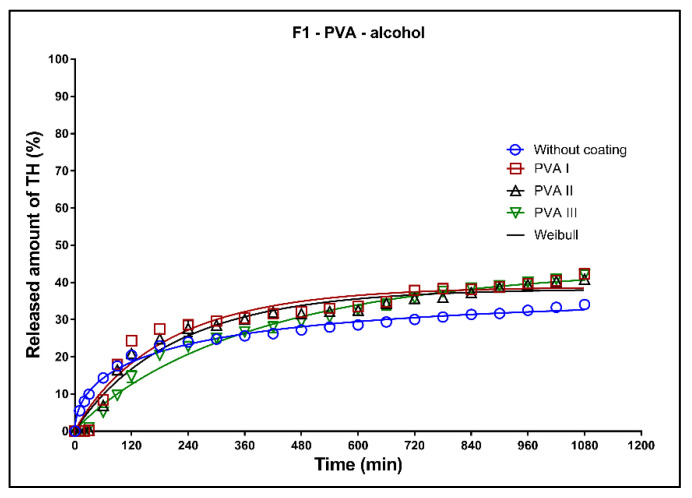
Dissolution profiles of F1 formulation in alcoholic medium (40% of alcohol) fitted to the Weibull model: uncoated tablets and tablets with PVA coating (three values of the perimeter thickness).

**Figure 15:**
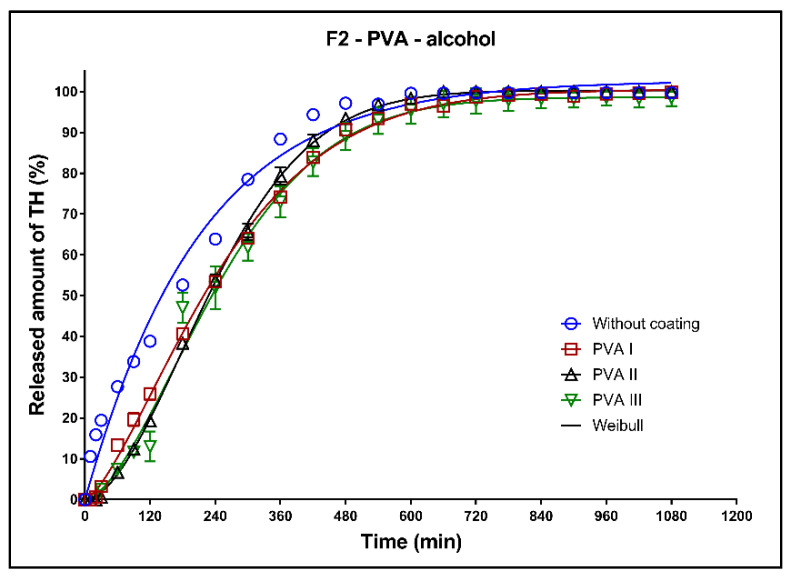
Dissolution profiles of F2 formulation in alcoholic medium (40% of alcohol) fitted to the Weibull model: uncoated tablets and tablets with PVA coating (three values of the perimeter thickness).

**Figure 16:**
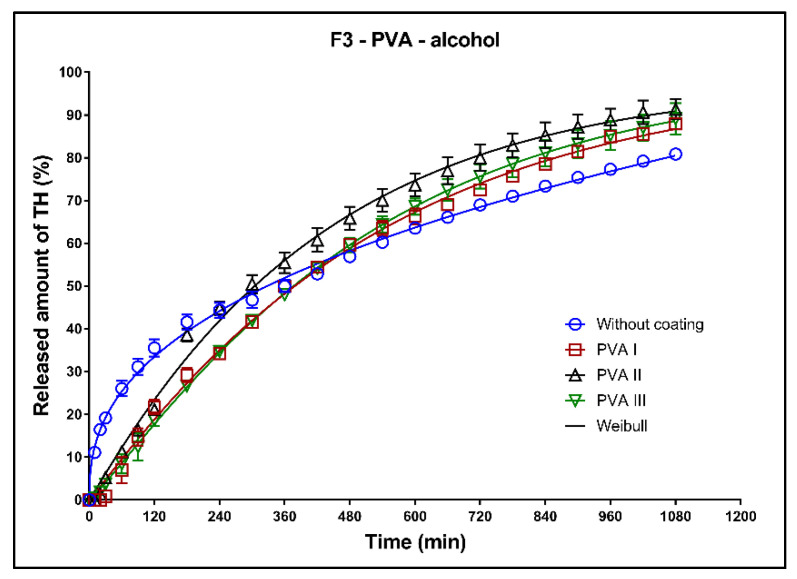
Dissolution profiles of F3 formulation in alcoholic medium (40% of alcohol) fitted to the Weibull model: uncoated tablets and tablets with PVA coating (three values of the perimeter thickness).

In Figure 14, Figure 15 and Figure 16, the dissolution profiles of the three studied formulations (F1–F3; see Table 1) without coating and with 3D-printed PVA coating (three values of perimeter thickness: PVA I, PVA II, PVA III) in alcoholic medium can be seen. The dissolution tests were performed for 2 h in alcoholic medium (40% of ethanol in acidic medium pH 1.2). After 2 h, the tablets were transferred to the non-alcoholic acidic medium and the dissolution testing continued for an additional 16 h as described in Section 2.2.8. The dissolution profiles were fitted to the first-order kinetic model and the Weibull model. The results of the non-linear regression analysis are summarized in Table 11, Table 12 and Table 13. A detailed description of the dissolution results follows.

**Formulation F1**: The maximum released amount of TH from the uncoated tablets (approx. 35%) was significantly reduced by the presence of ethanol, as can be seen in Figure 14. Due to the chemical interaction between the Kollidon SR and ethanol, a negative dose dumping effect was observed, and the release of the active substance was inhibited [8]. The coating caused a slight increase of the released TH amount and the fits for all three values of the perimeter thickness were the similar; see the coefficient *ß* in Table 11. 

**Formulation F2**: All dissolution profiles were fitted to the Weibull model. The maximum released amount of the model drug after 18 h was 100% for all tested tablets (Figure 15, Table 12). The release of the model drug from the uncoated tablets was faster during the first 6 h compared to the drug release from the tablets with coating. After 2 h of the dissolution in the alcoholic media, 40% of the model drug was released in case of the uncoated tablet, and 25, 20 and 15% of the model drug was released in case of the tablets coated with PVA I, PVA II, and PVA III perimeters, respectively.

**Formulation F3**: The fit of the dissolution profile of the coated tablets by the Weibull model was very similar, which was confirmed by the values of the parameter ß (Table 13). The coating increased the maximum released amount. The release of TH was faster for the uncoated tablets during the first 5 h, then (owing to the steeper dissolution profile) the release from the coated tablets became the faster of the two. 

#### 3.6.4. Alcoholic Medium (40% of Alcohol): Dissolution Tests of the Uncoated Tablets and Tablets with Affinisol Coating 

**Figure 17:**
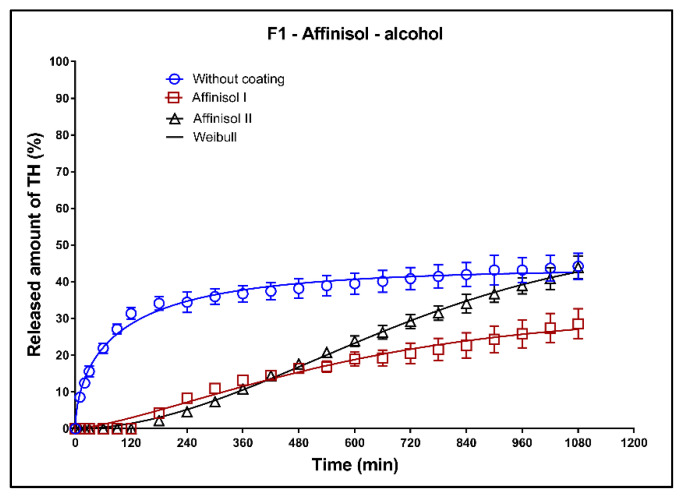
Dissolution profiles of F1 formulation in alcoholic medium (40% of alcohol) fitted to the Weibull model: uncoated tablets and tablets with Affinisol coating (two values of the perimeter thickness).

**Figure 18:**
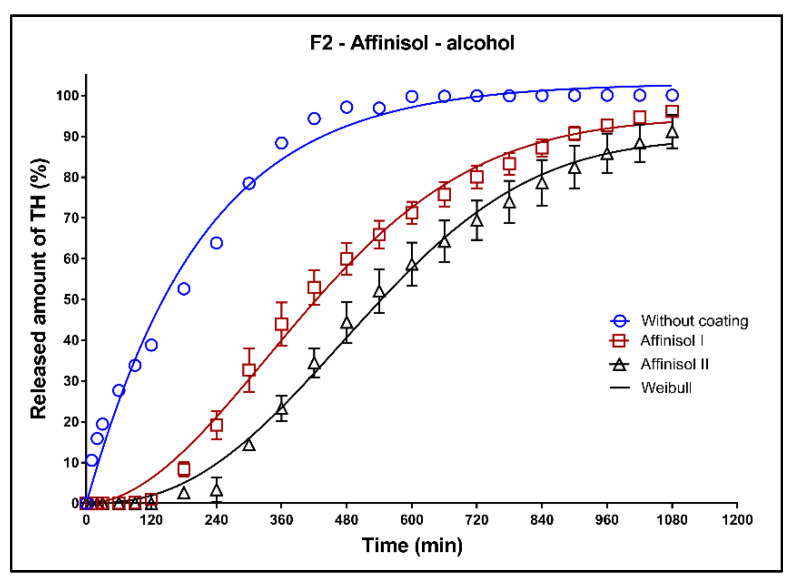
Dissolution profiles of F2 formulation in alcoholic medium (40% of alcohol) fitted to the Weibull model: uncoated tablets and tablets with Affinisol coating (two values of the perimeter thickness).

**Figure 19:**
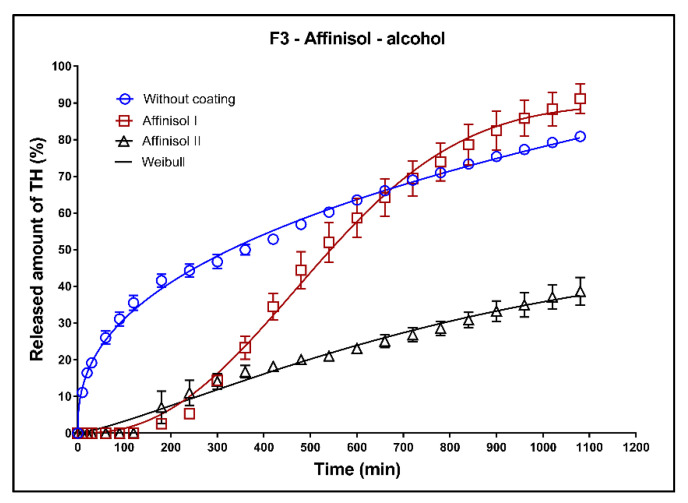
Dissolution profiles of F3 formulation in alcoholic medium (40% of alcohol) fitted to the Weibull model: uncoated tablets and tablets with Affinisol coating (two values of the perimeter thickness).

In Figure 17, Figure 18 and Figure 19, the dissolution profiles of the three studied formulations (F1–F3; see Table 1) without coating and with 3D-printed Affinisol coating (three values of thickness perimeters (AFF I, AFF II, AFF III)) in alcoholic medium can be seen. The dissolution tests were performed for 2 h in alcoholic medium (40% of ethanol in acidic medium pH 1.2). After 2 h, the tablets were transferred to the non-alcoholic acidic medium and the dissolution testing continued for an additional 16 h as described in Section 2.2.8. The dissolution profiles were fitted using the first-order kinetic model and the Weibull model. The results of the non-linear regression analysis are summarized in Table 14, Table 15 and Table 16. A detailed description of the dissolution results follows.

**Formulation F1**: The matrix tablets with AFF I and AFF II resisted the dissolution media for 2 h. The maximum released amount of TH after 18 h was 44% for the uncoated tablets. The tablets coated with 1 perimeter (AFF I) exhibited 90% of the total released amount of TH, whereas the AFF II tablets showed only 41% total release. The coating significantly changed the release mechanism of the matrix tablets. 

**Formulation F2**: Similarly to the F1 formulation, the AFF I and AFF II tablets were resistant against the dissolution media for 2 h (Figure 18). The release of TH from uncoated tablets follows the first-order kinetic, as can be seen from the value of the parameter ß (Table 15). The maximum released amount of TH after 18 h was 100% for the uncoated tablets. This amount decreased by approximately 5% for the AFF I tablets and by approximately 10% in case of the AFF II tablets. 

**Formulation F3**: Additionally, in the case of the F3 formulation, the coated tablets were resistant against the dissolution media for 2 h (Figure 19). The uncoated, AFF I, and AFF II tablets exhibited the maximum released TH amounts of 80, 90, and 37%, respectively. The dissolution profile of AFF II tablets exhibited a sigmoidal character, which confirmed the change of the mechanism of TH release compared to the uncoated tablets and AFF I tablets. On the other hand, the dissolution profile of AFF I tablets after 2 h of the dissolution was almost linear.

## 4. Conclusions

The tablets used for the extended drug release formulations commonly contain a large amount of drug and their release mechanism must be invariable to prevent any possibility of uncontrolled drug release, which can give rise to the dose dumping effect. The effect of the 3DP coatings with two types of coating materials (PVA, Affinisol) on the release mechanism and the release rate of the ionizing highly water-soluble drug (TH) from the hydrophilic (F1), lipophilic (F2), and hydrophilic-lipophilic (F3) matrix tablets was studied during dissolution tests in acidic and alcoholic dissolution media. The main results can be summarized as follows:

- The HME process was optimized for Affinisol, with the temperature of 150–160 °C being most effective.
- The procedures for 3D printing of Affinisol and PVA were optimized under the conditions given in Table 2.
- Based on the thermal and spectral analyses, no significant formation of the crystalline phase took place during the HME and 3D printing processes.
- The PVA coating did not influence the release mechanism of the model drug during the dissolution test in the acidic dissolution medium. The maximum released amount of the model drug from the formulation F1 (hydrophilic) was the same for the tablets without coating and for the tablets coated by one perimeter PVA. The coating by two and three perimeters of PVA decreased the maximum released amount of the model drug by ~10%. The maximum released amount of TH from the formulations F2 (lipophilic) and F3 (hydrophilic-lipophilic) was not influenced by the PVA coating.
- The PVA coating did not influence the release mechanism of the model drug in the alcoholic dissolution medium. The presence of Kollidon^®^ SR caused the negative dose dumping effect and the release of the model drug was inhibited. The PVA coating did not prevent the release of the model drug during the first two hours of the dissolution testing in the alcoholic dissolution media.
- The Affinisol coating changed the release mechanism of the model drug in the acidic dissolution media. The coating reduced the model drug release at the beginning of the dissolution test.
- The Affinisol coating also changed the release mechanism of the model drug in the alcoholic dissolution media. At the beginning of the dissolution tests, no model drug was released for a significant time. Additionally, for the AFF-coated tablets, the Kollidon^®^ SR caused the negative dose dumping effect. The Affinisol coating was, however, found to be suitable for the prevention of the dose dumping effect.

In conclusion, the Affinisol coating was found to be markedly more beneficial due to its impact on the release rate of the active substance. The dissolution tests in the alcoholic medium showed that the Affinisol coating was effective in preventing the dose dumping incidence. The dissolution tests in the acidic dissolution media showed that the Affinisol coating can also be useful for the delayed release of active substances. The hydrophilic-lipophilic formulation F3, which is a combination of Kollidor SR and Compritol, was found to be most suitable for usage with the Affinisol coating. The results of the present case study will be further extended in the consequent paper on the topic of the HME and 3D printing in pharmacy. So far, only a limited number of dosage forms that resist the effect of alcohol are available. The present study introduces (as a case study) the detailed development of such a dosage form based on the 3D-printed coating. 

The 3D coating process is a non-solvent method that is very important in many aspects. Once the filament is prepared by the HME process, there is no need for other chemicals, such as plasticizers, antiadhesives, surfactants, and pigments, which increase the chemical variability and lower the reproducibility. Hence, overall, the 3D coating method is less complex. The 3D coating method is also environmentally sustainable: 3D printing creates coatings using a waste-free procedure, where any unsuccessful prints can be recycled by re-extrusion, also saving costs. Due to its variability, inherently low-mass production, and easy-to-adapt nature, 3D coating represents an interesting tool for implementation in personalized medicine. Moreover, the 3D coating method is also advantageous due to it being suitable for fragile tablets. Matrices mechanically nonresistant to abrasion can also be coated by this method, in contrast with the commercial film coating method. 

Note that a follow-up study is under preparation, focusing on the development of tailored pharmaceutical filaments usable in the 3D coating process.

## Figures and Tables

**Table 1:** Compositions of the studied formulations (%).

Formulation	F1	F2	F3
Tramadol hydrochloride	20	20	20
Kollidon^®^ SR	50	-	25
Compritol^®^ 888 ATO	-	50	25
Kolliwax^®^ S	5	5	5
Prosolv^®^ SMCC 90	25	25	25

**Table 2:** Summary of the thermal parameters for successful 3D printing from AFF and PVA.

Material	Nozzle Temperature	Bed Temperature	Notes
AFF	205 °C	95 °C	Very fragile imprints. Requires cautious removal from the bed. Lower temperature causes worse cohesion of the layers.
PVA	190 °C	50–80 °C	Higher temperature of the nozzle causes shape deformation

**Table 3:** Three-dimensional coating times depending on the number of coatings and their thickness (perimeters) for the whole coating process. The final step (after inserting the tablet) takes approximately 25% of the listed times.

3D Coating Times			
Number of Coatings	Thickness P1	Thickness P2	Thickness P3
1	3 min	3 min	4 min
7	19 min	23 min	30 min
10	27 min	32 min	42 min
50	133 min	159 min	208 min

**Table 4:** Non-linear regression analysis of the dissolution profiles of F1 formulation in acidic medium: uncoated tablets and tablets with PVA coating (three values of the perimeter thickness).

	Weibull Model				
	(*k*_w_ ± *SD*) × 10^3^ (min^−ß^)	*A*_∞_ ± *SD* (%)	*ß* ± *SD*	*ASS*	*R* ^2^
Without coating	25.57 ± 1.57	113.6 ± 2.89	0.63 ± 0.01	112	0.9974
PVA I	29.06 ± 2.13	107.8 ± 2.84	0.62 ± 0.02	153	0.9962
PVA II	0.42 ± 0.12	91.9 ± 1.98	1.29 ± 0.05	311	0.9940
PVA III	0.04 ± 0.01	85.3 ± 1.25	1.69 ± 0.07	313	0.9940

**Table 5:** Non-linear regression analysis (the first-order kinetic model) of the dissolution profiles of F2 formulation in acidic medium: uncoated tablets and tablets with PVA coating (three values of the perimeter thickness).

	The First-Order Kinetic Model			
	(*k*_1_ ± *SD*) × 10^3^ (min^−1^)	*A*_∞_ ± *SD* (%)	*ASS*	*R* ^2^
Without coating	5.84 ± 0.2	103.6 ± 0.99	726	0.9865
PVA I	2.94 ± 0.1	109.9 ± 1.87	708	0.9900
PVA II	5.75 ± 0.2	114.1 ± 2.88	1400	0.9820
PVA III	2.82 ± 0.2	113.9 ± 3.74	2507	0.9691

**Table 6:** Non-linear regression analysis (Weibull model) of the dissolution profiles of F2 formulation in acidic medium: uncoated tablets and tablets with PVA coating (three values of the perimeter thickness).

	Weibull Model				
	(*k*_w_ ± *SD*) × 10^3^ (min^−ß^)	*A*_∞_ ± *SD* (%)	*ß* ± *SD*	*ASS*	*R* ^2^
Without coating	14.37 ± 2.10	107.1 ± 1.43	0.81 ± 0.03	436	0.9919
PVA I	0.61 ± 0.10	102.3 ± 0.79	1.30 ± 0.03	203	0.9971
PVA II	0.19 ± 0.05	102.7 ± 0.67	1.51 ± 0.04	213	0.9973
PVA III	0.05 ± 0.01	101.6 ± 0.54	1.78 ± 0.05	193	0.9976

**Table 7:** Non-linear regression analysis of the dissolution profiles of F3 formulation in acidic medium: uncoated tablets and tablets with PVA coating (three values of the perimeter thickness).

	Weibull Model				
	(*k*_w_ ± *SD*) × 10^3^ (min^−ß^)	*A*_∞_ ± *SD* (%)	*ß* ± *SD*	*ASS*	*R* ^2^
Without coating	23.28 ± 2.1	100.4 ± 2.19	0.68 ± 0.02	204	0.9957
PVA I	0.87 ± 0.2	99.4 ± 0.92	1.23 ± 0.03	201	0.9969
PVA II	0.21 ± 0.6	99.0 ± 0.91	1.46 ± 0.04	253	0.9964
PVA III	0.22 ± 0.04	99.3 ± 0.71	1.46 ± 0.03	162	0.9976

**Table 8:** Non-linear regression analysis of the dissolution profiles of F1 formulation in acidic medium: uncoated tablets and tablets with Affinisol coating (two values of the perimeter thickness).

	Weibull Model				
	(*k*_w_ ± *SD*) × 10^3^ (min^−ß^)	*A*_∞_ ± *SD* (%)	*ß* ± *SD*	*ASS*	*R* ^2^
Without coating	25.57 ± 1.57	113.6 ± 2.89	0.63 ± 0.01	112	0.9974
Affinisol I	1.47 ± 0.02	94.5 ± 1.74	3.28 ± 0.14	365	0.9939
Affinisol II	1.48 ± 0.03	75.5 ± 1.57	3.38 ± 0.18	357	0.9911

**Table 9:** Non-linear regression analysis of the dissolution profiles of F2 formulation in acidic medium: uncoated tablets and tablets with Affinisol coating (two values of the perimeter thickness).

	Weibull Model				
	(*k*_w_ ± *SD*) × 10^3^ (min^−ß^)	*A*_∞_ ± *SD* (%)	*ß* ± *SD*	*ASS*	*R* ^2^
Without coating	14.37 ± 2.10	107.1 ± 1.43	0.89 ± 0.03	436	0.9919
Affinisol I	0.02 ± 0.01	93.9 ± 1.97	1.75 ± 0.09	595	0.9905
Affinisol II	0.02 ± 0.01	91.2 ± 1.89	2.09 ± 0.12	613	0.9899

**Table 10:** Non-linear regression analysis of the dissolution profiles of F3 formulation in acidic medium: uncoated tablets and tablets with Affinisol coating (two values of the perimeter thickness).

	Weibull Model				
	(*k*_w_ ± *SD*) × 10^3^ (min^−ß^)	*A*_∞_ ± *SD* (%)	*ß* ± *SD*	*ASS*	*R* ^2^
Without coating	23.28 ± 2.1	100.4 ± 2.19	0.68 ± 0.02	204	0.9957
Affinisol I	2.16 ± 0.04	95.6 ± 1.19	1.97 ± 0.08	338	0.9951
Affinisol II	1.77 ± 0,02	91.5 ± 1.01	2.33 ± 0.06	153	0.9974

**Table 11:** Non-linear regression analysis of the dissolution profiles of F1 formulation in alcoholic medium (40% of alcohol) fitted to the Weibull model: uncoated tablets and tablets with PVA coating (three values of the perimeter thickness).

	Weibull Model				
	(*k*_w_ ± *SD*) × 10^3^ (min^−ß^)	*A*_∞_ ± *SD* (%)	*ß* ± *SD*	*ASS*	*R* ^2^
Without coating	59.07 ± 5.1	36.62 ± 1.45	0.52 ± 0.03	38	0.9907
PVA I	7.62 ± 3.31	38.86 ± 1.14	0.92 ± 0.09	388	0.9580
PVA II	6.12 ± 2.44	38.34 ± 1.06	0.95 ± 0.08	289	0.9679
PVA III	4.23 ± 1.18	44.41 ± 2.15	0.91 ± 0.06	137	0.9858

**Table 12:** Non-linear regression analysis of the dissolution profiles of F2 formulation in alcoholic medium (40% of alcohol) fitted to the Weibull model: uncoated tablets and tablets with PVA coating (three values of the perimeter thickness).

	Weibull Model				
	(*k*_w_ ± *SD*) × 10^3^ (min^−ß^)	*A*_∞_ ± SD (%)	*ß* ± *SD*	*ASS*	*R* ^2^
Without coating	4.37 ± 1.06	102.7 ± 1.41	1.02 ± 0.05	683	0.9881
PVA I	0.32 ± 0.05	100.5 ± 0.45	1.43 ± 0.03	94	0.9987
PVA II	0.04 ± 0.05	100.3 ± 0.25	1.78 ± 0.02	42	0.9995
PVA III	0.09 ± 0.04	98.7 ± 0.98	1.64 ± 0.07	560	0.9925

**Table 13:** Non-linear regression analysis of the dissolution profiles of F3 formulation in alcoholic medium (40% of alcohol) fitted to the Weibull model: uncoated tablets and tablets with PVA coating (three values of the perimeter thickness).

	Weibull Model				
	(*k*_w_ ± *SD*) × 10^3^ (min^−ß^)	*A*_∞_ ± *SD* (%)	*ß* ± *SD*	*ASS*	*R* ^2^
Without coating	1.81 ± 0.04	99.9 ± 0.15	0.58 ± 0.02	306	0.9875
PVA I	1.39 ± 0.25	98.3 ± 4.17	1.05 ± 0.04	63	0.9971
PVA II	1.96 ± 0.38	97.9 ± 2.75	1.03 ± 0.04	65	0.9973
PVA III	0.96 ± 0.15	98.7 ± 0.88	1.11 ± 0.01	4	0.9998

**Table 14:** Non-linear regression analysis of the dissolution profiles of F1 formulation in alcoholic medium (40% of alcohol) fitted to the Weibull model: uncoated tablets and tablets with Affinisol coating (two values of the perimeter thickness).

	Weibull Model				
	(*k*_w_ ± *SD*) × 10^3^ (min^−ß^)	*A*_∞_ ± *SD* (%)	*ß* ± *SD*	*ASS*	*R* ^2^
Without coating	64.67 ± 11.7	43.51 ± 1.14	0.59 ± 0.05	214	0.9695
Affinisol I	0.14 ± 0.12	30.73 ± 3.49	1.38 ± 0.16	170	0.9648
Affinisol II	0.003 ± 0.02	50.29 ± 3.05	1.89 ± 0.10	70	0.9937

**Table 15:** Non-linear regression analysis of the dissolution profiles of F2 formulation in alcoholic medium (40% of alcohol) fitted to the Weibull model: uncoated tablets and tablets with Affinisol coating (two values of the perimeter thickness).

	Weibull Model				
	(*k*_w_ ± *SD*) × 10^3^ (min^−ß^)	*A*_∞_ ± *SD* (%)	*ß* ± *SD*	*ASS*	*R* ^2^
Without coating	4.42 ± 1.06	103.1 ± 1.42	1.01 ± 0.05	677	0.9883
Affinisol I	0.007 ± 0.004	94.6 ± 1.64	1.91 ± 0.09	433	0.9933
Affinisol II	0.002 ± 0.001	89.5 ± 2.10	2.39 ± 0.13	535	0.9906

**Table 16:** Non-linear regression analysis of the dissolution profiles of F3 formulation in alcoholic medium (40% of alcohol) fitted to the Weibull model: uncoated tablets and tablets with Affinisol coating (two values of the perimeter thickness).

	Weibull Model				
	(*k*_w_ ± *SD*) × 10^3^ (min^−ß^)	*A*_∞_ ± *SD* (%)	*ß* ± *SD*	*ASS*	*R* ^2^
Without coating	23.28± 2.05	110.4 ± 2.19	0.68 ± 0.02	204	0.9957
Affinisol I	0.006 ± 0.002	95.6 ± 1.19	1.97 ± 0.08	338	0.9951
Affinisol II	0.004 ± 0.001	91.53 ± 1.01	2.33 ± 0.06	153	0.9974

## Data Availability

A full list of references is compiled and attached to this manuscript.

## Supplementary Materials

The following are available online at https://www.mdpi.com/article/10.3390/pharmaceutics13122123/s1, Figure S1: Dissolution profiles of F1 formulation at pH 6.8 fitted to the Weibull model—uncoated tablets and tablets with Affinisol coating (one perimeter thickness). Figure S2: Dissolution profiles of F2 formulation in pH 6.8 medium fitted to the Weibull model—uncoated tablets and tablets with Affinisol coating (one perimeter thickness). Figure S3: Dissolution profiles of F3 formulation in pH 6.8 medium fitted to the Weibull model—uncoated tablets and tablets with Affinisol coating (one perimeter thickness). Table S1: Non-linear regression analysis of the dissolution profiles of F1 formulation in pH 6.8 medium—uncoated tablets and tablets with Affinisol coating (one perimeter thickness). Table S2: Non-linear regression analysis of the dissolution profiles of F2 formulation in pH 6.8 medium—uncoated tablets and tablets with Affinisol coating (one perimeter thickness). Table S3: Non-linear regression analysis of the dissolution profiles of F3 formulation in pH 6.8 medium—uncoated tablets and tablets with Affinisol coating (one perimeter thickness).

## Abbreviations

3DP	Three-dimensional printing
ADD	Alcohol-induced dose dumping
AFF	Affinisol
API	Active pharmaceutical substance
ASS	Absolute sum of squares
DSC	Differential scanning calorimetry
FDA	Food and Drug Administration
HME	Hot-melt extrusion
HPMC	Hypromellose
P	Perimeters
PE	Polyethylene
PVA	Polyvinyl alcohol
PXRD	Powder X-ray diffraction
SD	Standard deviation
SEM	Scanning electron microscopy
TH	Tramadol hydrochloride
