# Supplementary material for: 3D-Printed Coating of Extended-Release Matrix Tablets: Effective Tool for Prevention of Alcohol-Induced Dose Dumping Effect
Source: Pharmaceutics. 2021 Dec 9;13(12):2123. doi: 10.3390/pharmaceutics13122123 (PMC8705548; doi:10.3390/pharmaceutics13122123)
Supplement: Supplementary file 1 [file pharmaceutics-13-02123-s001.zip › pharmaceutics-1477562-supplementary.pdf]

pharmaceutics MDPI

# Supplementary data – disolution tests at pH 6.8

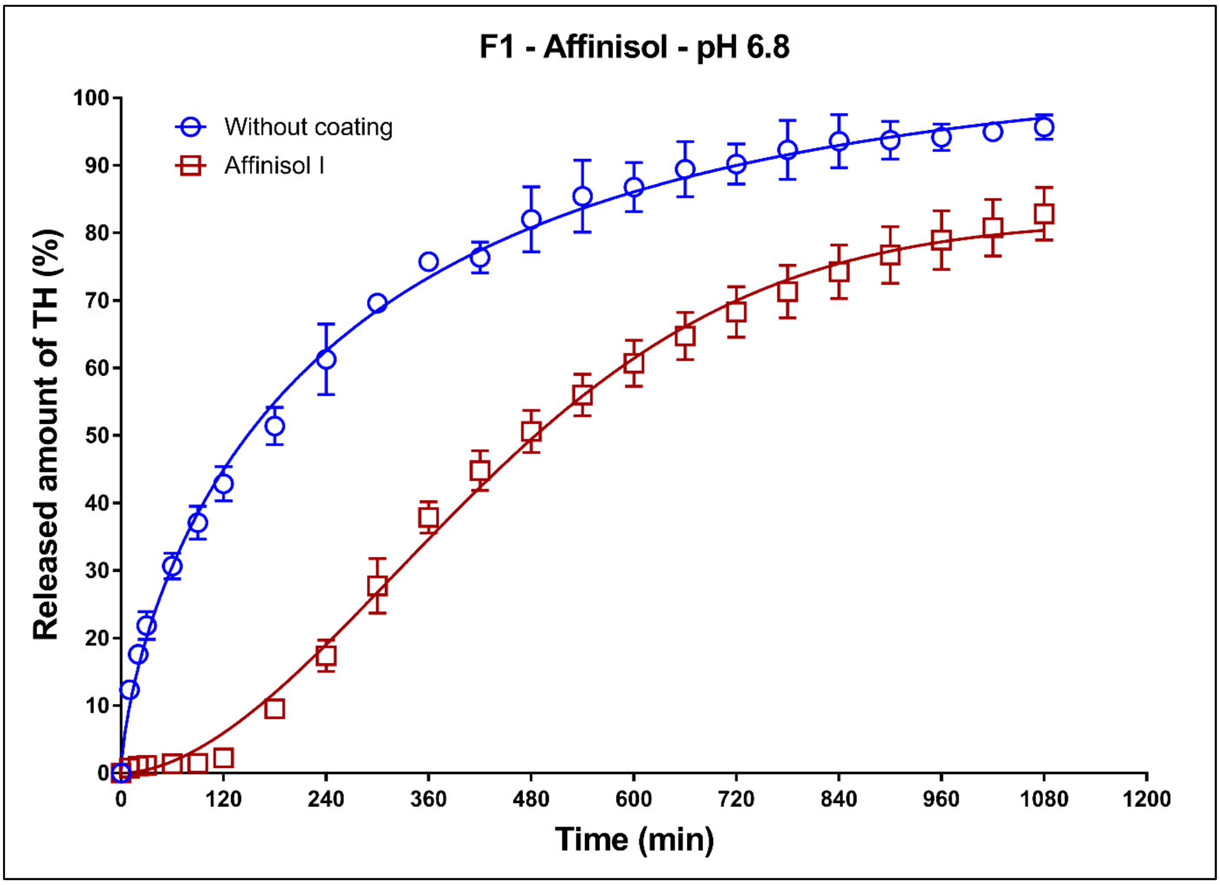


**Figure S1**: Dissolution profiles of F1 formulation at pH 6.8 fitted to the Weibull model – uncoated tablets and tablets with Affinisol coating (one perimeter thickness).

**Table S1:** Non-linear regression analysis of the dissolution profiles of F1 formulation in pH 6.8 medium – uncoated tablets and tablets with Affinisol coating (one perimeter thickness).

| | Weibull model | | | | |
|---|---|---|---|---|---|
| | ($k_w \pm SD$) x $10^3$ ($min^{-ß}$) | $A_\infty \pm SD$ (%) | $ß \pm SD$ | *ASS* | $R^2$ |
| Without coating | 19.93 ± 2.4 | 105 ± 2.92 | 0.69 ± 0.03 | 314 | 0.9927 |
| Affinisol I | 0.01 ± 0.006 | 81.88 ± 1.63 | 1.81 ± 0.08 | 323 | 0.9930 |

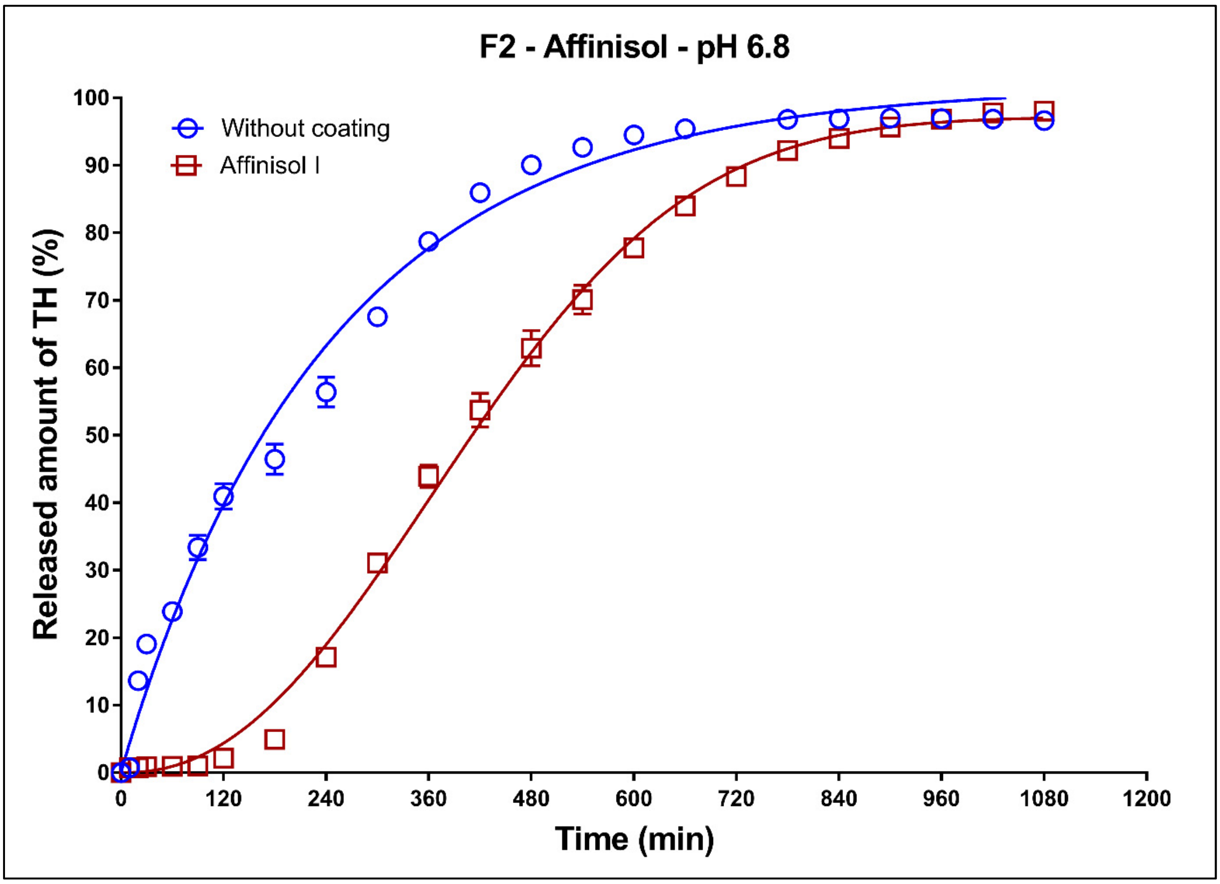


**Figure S2**: Dissolution profiles of F2 formulation in pH 6.8 medium fitted to the Weibull model – uncoated tablets and tablets with Affinisol coating (one perimeter thickness).

**Table S2:** Non-linear regression analysis of the dissolution profiles of F2 formulation in pH 6.8 medium – uncoated tablets and tablets with Affinisol coating (one perimeter thickness).

| | Weibull model | | | | |
|---|---|---|---|---|---|
| | $(k_w \pm SD)$ x $10^3$ ($min^{-ß}$) | $A_\infty \pm SD$ (%) | $ß \pm SD$ | *ASS* | $R^2$ |
| Without coating | 4.68 ± 1.3 | 101.8 ± 2.08 | 0.97 ± 0.05 | 840 | 0.9852 |
| Affinisol I | 0.001 ± 0.003 | 97.2 ± 0.74 | 2.23 ± 0.06 | 166 | 0.9933 |

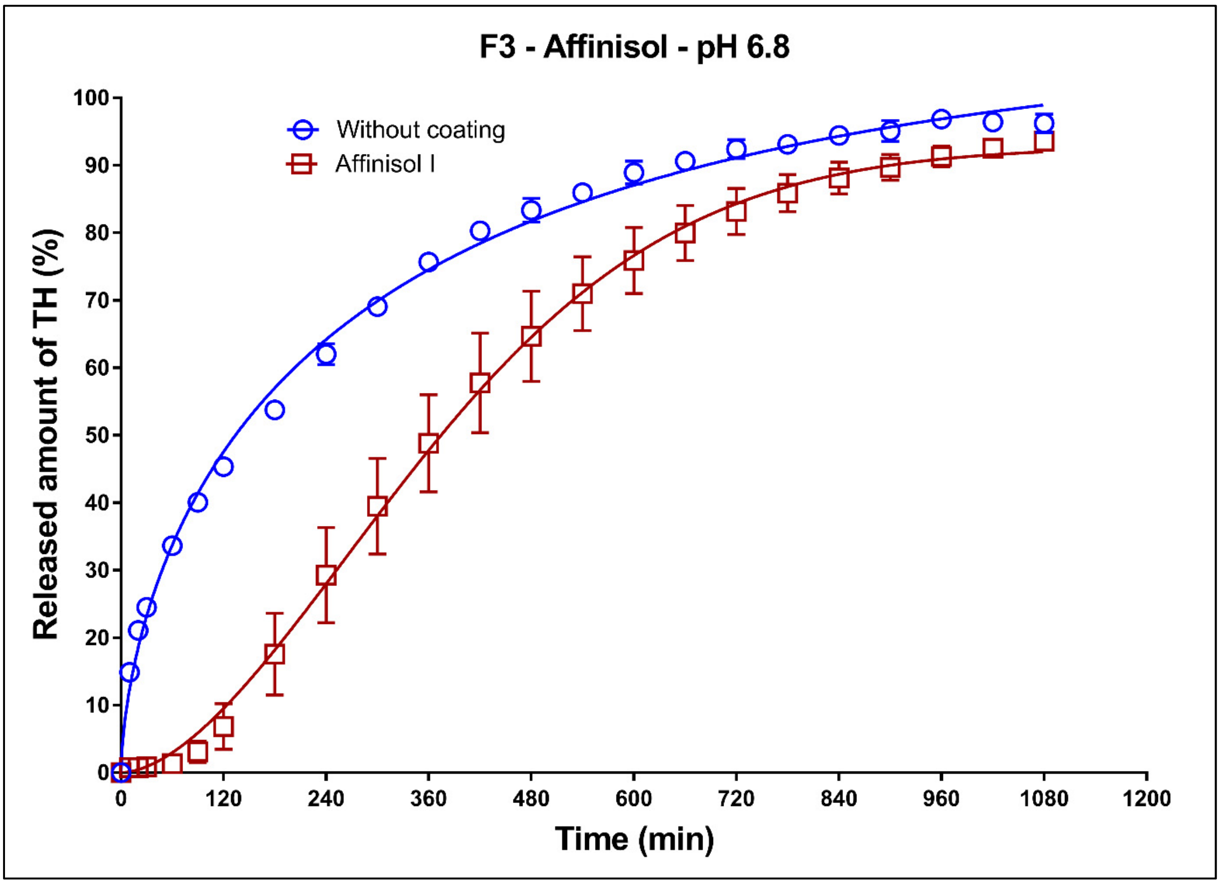


**Figure S3**: Dissolution profiles of F3 formulation in pH 6.8 medium fitted to the Weibull model – uncoated tablets and tablets with Affinisol coating (one perimeter thickness).

**Table S3:** Non-linear regression analysis of the dissolution profiles of F3 formulation in pH 6.8 medium – uncoated tablets and tablets with Affinisol coating (one perimeter thickness).

| | Weibull model | | | | |
|---|---|---|---|---|---|
| | ($k_w$ ± *SD*) x $10^3$ (min$^{-ß}$) | $A_\infty$ ± *SD* (%) | *ß* ± *SD* | *ASS* | $R^2$ |
| Without coating | 27.9 ± 2.04 | 111.2 ± 2.87 | 0,63 ± 0.02 | 158 | 0.9963 |
| Affinisol I | 0.028 ± 0.01 | 92.7 ± 1.51 | 1.73 ± 0.08 | 471 | 0.9924 |
